# Alternative splicing signatures in preimplantation embryo development

**DOI:** 10.1186/s13578-020-00399-y

**Published:** 2020-03-10

**Authors:** Geng G. Tian, Jing Li, Ji Wu

**Affiliations:** 1 Renji Hospital, Key Laboratory for the Genetics of Developmental & Neuropsychiatric Disorders (Ministry of Education), Bio-X Institutes, School of Medicine, Shanghai Jiao Tong University, Shanghai, 200240 China; 2 Key Laboratory of Fertility Preservation and Maintenance of Ministry of Education, NingxiaMedicalUniversity, Yinchuan, 750004 China; 3 Department of Bioinformatics and Biostatistics, School of Life Sciences and Biotechnology, Shanghai Jiao Tong University, 800 Dongchuan Road, Shanghai, 200240 China; 4 Shanghai Key Laboratory of Reproductive Medicine, Shanghai, 200025 China

## Abstract

**Background:**

Preimplantation embryo development is a highly ordered sequence of processes and it requires a precise temporal and spatial control of gene expression. Alternative splicing (AS) is a crucial process that changes the genomic instructions into functional proteins, playing a critical role in the regulation of gene expression. Therefore, studies of AS can significantly improve our understanding of transcription and splicing events in preimplantation embryo development.

**Results:**

To study signatures of AS in embryo development, we firstly identified the critical stage for gene transcription during the preimplantation embryo development. By analyzing single cells RNA-seq (scRNA-seq) data, we found that the two-cell stage is a critical stage for gene transcription in preimplantation embryo development. Further study showed that AS was widespread in preimplantation embryo development, especially at the two-cell stage. In combination with high-throughput chromosome conformation (Hi-C) data, we demonstrated that AS genes were highly enriched in TAD boundaries, while they had no relationship with the A/B compartment and TAD.

**Conclusion:**

Our results provide new insight into the relationship among AS, gene transcription and chromatin structure in preimplantation embryo development.

## Introduction

Embryogenesis is a sequential series of dynamic processes including cell division and growth, and the elaboration of differentiation programs leading to cell fate specification. Preimplantation embryo development plays a crucial role in fetal development and requires a precise temporal and spatial control of gene expression. Specifically, profiling of the gene expression in preimplantation embryo development can show when and how cell fate decisions are made. Initially, RNA-seq could not be applied to the study of preimplantation embryo development because of its technological limitations. However, through the development of RNA-seq technology for a low input of cells, such as Cel-seq [1], Smart-seq [2], and RAGE-seq [3], our understanding of the gene expression of small cell populations has been greatly extended. Recently, single-cell RNA-seq (scRNA-seq) techniques including chromium 10× [4] and Drop-seq [5] have been developed, which can detect the gene expression in just a single cell; these approaches have significantly advanced our knowledge of cell systems. These new technologies have also greatly facilitated the study of preimplantation embryo development. For example, Tang’s group made major progress in understanding preimplantation embryo development [6, 7] by profiling the landscape of gene expression during this process. At the transcriptome level, one of the most important types of pre-mRNA processing is alternative splicing (AS) events, which can increase the diversity of the transcriptome and proteome [8] and play an important role in the regulation of gene expression in a variety of eukaryotes. Approximately 95% of multi-exon genes in human undergo AS. Misregulation of AS leads to splicing defects causing severe diseases. Therefore, studies of alternative splicing can significantly improve our understanding of transcription and splicing events in embryo development. Although previous studies reported that AS was specific to different tissues and cell types [9], it remains unclear when and how AS occurs in preimplantation embryo development.

Concurrent with this progress in structure variation, Hi-C was developed to clarify chromatin organization [10]. To better understand the relationship between transcription and chromatin structure in preimplantation embryo development, Xie and Liu’s group developed the small-cell Hi-C technology (scHi-C) [11, 12] and revealed that the high-order chromatin structure underwent reprogramming, in which the topologically associating domains (TADs) disappeared in the MII and zygote stages, while TADs started to construct at the two-cell stage and completed at the eight-cell stage. Moreover, they used RNA-seq data to systematically analyze the relationship between gene expression and chromatin organization, but they did not consider genes whose products varied due to AS events. Therefore, it has remained unknown whether AS events are related to the high-order chromatin organization.

## Methods

### RNA-seq data analysis

RNA-seq data were downloaded from the online version of a previous paper [13]. The raw data were trimmed with BBmap (version 38.16) to obtain clean data, which had a quality score higher than 15 (Q > 15), followed by removal of the adaptors of those reads. Then, the reads were mapped to the mouse reference genome (mm9) with STAR (version 2.7.1a) [14] software using two-pass mode with the GENCODE annotation file (version Mouse Release M1). Gene expression (FPKM) levels were calculated using Cufflinks (version v2.2.1) [15]. Then, the gene expression was clustered by the c-means method to analyze the time course of gene expression, which was supported by the MFuzz R package [16]. The counts of gene expression were quantified by Rsubread software (version 1.6.2) [17]. DESeq2 package (version 1.10.1) [18] was used to identify the differential gene expression with the following cut-offs: FDR < 0.05 and log2FC > 2.

### Differential splicing and alternative splicing identification

Gene products encoded by different numbers of exons of the source gene were identified using the DEXSeq R package [19] with FDR < 0.05. Then, rMATS (version v4.0.3beta) was used to identify the different types of AS events with the splice junction file (SJ.out.tab) generated by STAR and two replicate bam files. According to the rMATS results, normalized percent spliced-in (PSI) values were calculated to identify the different AS events (FDR < 0.05).The AS genes were visualized with DEXseq and rmats2sashimiplot.

### Protein–protein network

AS gene interactions were input into STRING (version 11.0) [20] to identify the protein–protein network with the highest confidence (> 0.9). The network result was then displayed using Gephi software (version 0.9.2) [21].

### Gene ontology (GO) analysis

GO term enrichment analysis was performed using the Gene Ontology website (https://geneontology.org/, version 14) [22]. We chose an FDR threshold (Benjamini-corrected p value)of less than 0.05 to represent significance.

### Hi-C data analysis

Paired-end clean reads of Hi-C were process as previous described using HiCPro (version 2.7) [11, 23]. Briefly, sequencing reads were aligned to the mouse reference genome (mm9) and removed the re-ligation reads, continuous reads and PCR artifacts. ICM [24] normalization were used to remove the bias in the Hi-C data. HiTC [25] was used to calculate the A/B compartment status in 400 kb resolution with the options: normPerExpected = TRUE, npc = 1, which positive value represented A compartment while negative value represented B compartment. TAD was called with the directional index as previously described [26] in 20 kb resolution. While TAD boundary was defined as the nearby region smaller than 400 kb between two TADs.

## Result

### The two-cell stage is a critical stage for gene transcription in preimplantation embryo development

**Fig. 1:**
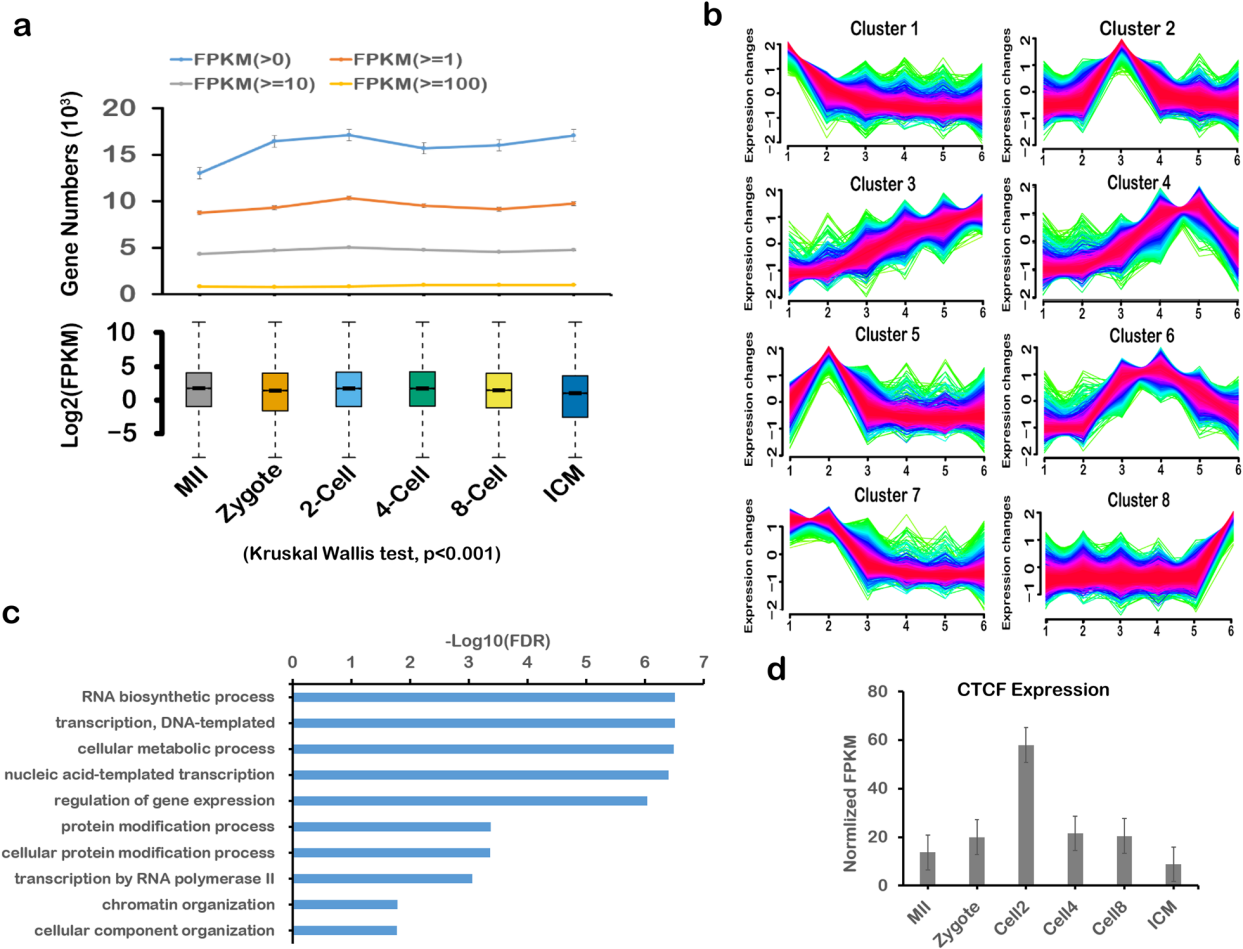
Transcriptome analysis in preimplantation embryo development. **a** Numbers and expressions in preimplantation embryo development. **b** Clusters of gene expression in preimplantation embryo development. **c** Gene Ontology enriched pathways in cluster 2 genes. **d** CTCF expression in RNA-seq data

AS is a process for enabling a messenger RNA (mRNA) to direct synthesis of different protein variants (isoforms) and plays a critical role in the regulation of gene expression. To study signatures of AS in preimplantation embryo development, we firstly identified the critical stage for gene transcription during the embryo development. Gene expression profiling of preimplantation embryo development was performed using RNA-seq data obtained from the study by Xie et al. [27], including MII oocytes, pronuclear stage 5 (PN5) zygotes, two-cell, four-cell, and eight-cell embryos, and inner cell masses (ICMs) from blastocysts. We respectively identified 13,026, 16,345, 17,117, 15,699, 16,012, and 17,080 expressed genes (FPKM > 0) in these stages using the STAR software [14], followed by its two-pass mode (Fig. 1a). Meanwhile, according to the gene expression (FPKM), we identified genes differentially expressed in preimplantation embryo development (Kruskal–Wallis test, p < 0.001) (Fig. 1a). The results showed that genes more highly expressed in FPKM ≥ 10did not change markedly, while genes expression at lower levels in FPKM < 10 significantly fluctuated, with the two-cell stage having the highest number of expressed genes (FPKM > 1). These findings suggest that the two-cell stage is a critical stage at which most genes with lower expression (FPKM < 10) started to be transcribed during preimplantation embryo development.

To identify those genes specifically expressed in the two-cell stage, we used the fuzzy c-means (FCM) method to perform a time-course analysis of RNA-seq data from preimplantation embryo development. We divided the expressed genes into eight clusters and discovered 1415 genes in cluster 2, representing the genes specifically expressed in the two-cell stage (Fig. 1b). Gene Ontology enrichment showed that these genes were highly related to RNA transcription and gene expression, and interestingly also to chromatin organization (Fig. 1c). In line with a previous report describing that the two-cell stage is the point at which the construction of topologically associating domains (TADs) is initiated in chromatin organization [11, 12, 26], we found that *CTCF* (CCCTC-binding factor) was also included in cluster 2, which is a factor that was reported with the formation of TAD and chromatin loops [28], and highly expressed in the two-cell stage (Fig. 1d). Taken together, these results show that the two-cell stage is critical stage for gene transcription during preimplantation embryo development.

### Dynamic changes of alternative splicing in preimplantation embryo development

**Fig. 2:**
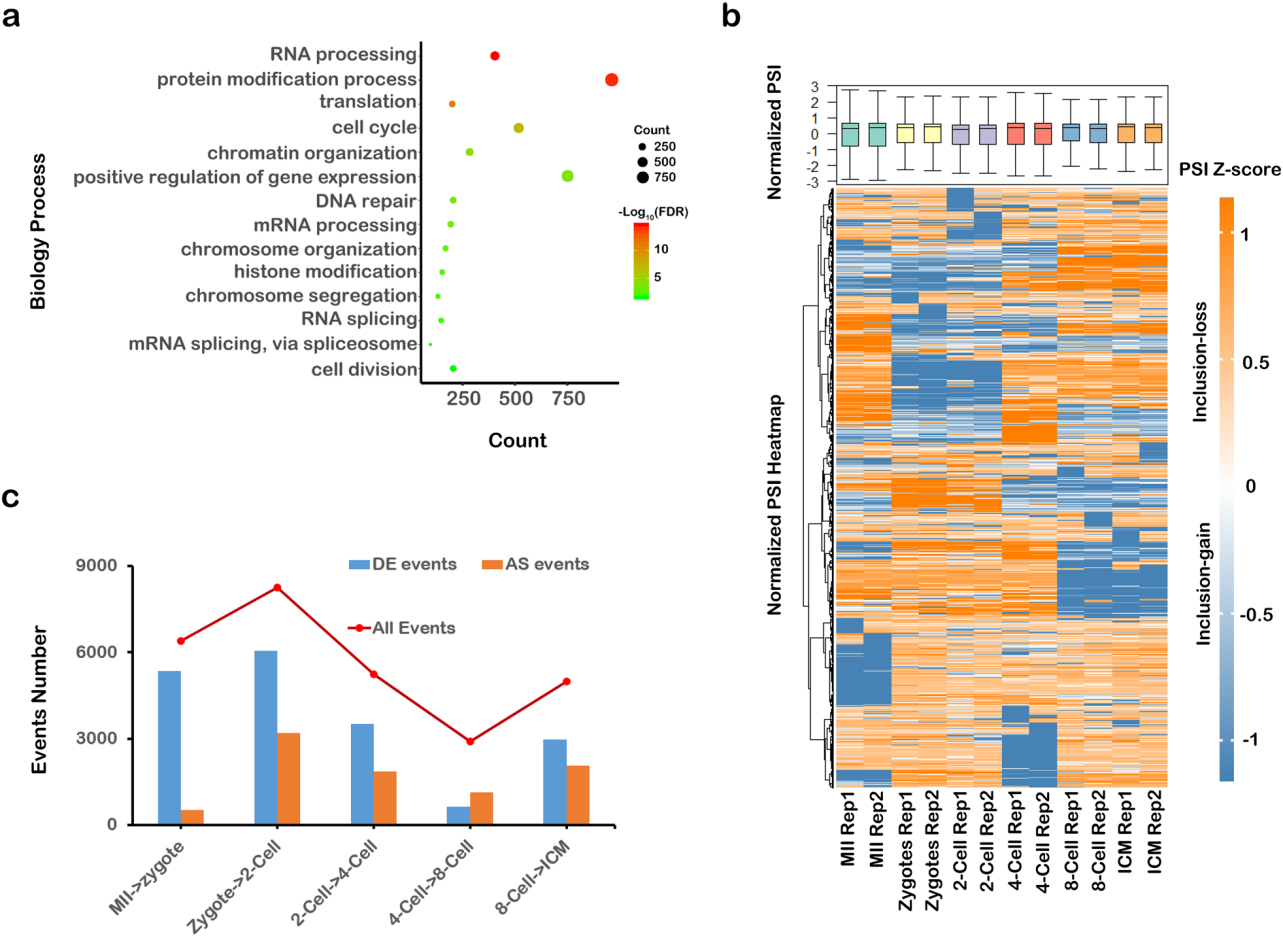
Alternative splicing in preimplantation embryo development. **a** Gene Ontology pathways of genes with significantly different expressed between zygotes and the two-cell stage. **b** A heatmap of normalized PSI values in preimplantation embryo development. **c** The numbers of DE and AS events in preimplantation embryo development

Next, we found that the genes with a significant change of expression in the two-cell stage compared with their expression in zygotes were particularly associated with RNA splicing (Fig. 2a). It is thus suggested that RNA alternative slicing may play an important role in preimplantation embryo development. To characterize the AS events occurring during preimplantation embryo development, we used percent spliced-in (PSI) to measure the proportion of reads that aligned to splice junctions, which can support the inclusion isoforms [29]. Thousands of AS events were identified according to their PSI, and we divided the genes according to their PSI changes as inclusion-loss and inclusion-gain groups (Fig. 2b). The heatmap showed that PSI dynamically changed in preimplantation embryo development, suggesting that AS events are widespread in preimplantation embryo development. Furthermore, we screened the AS events in preimplantation embryo development and detected 6391 events (MII/zygotes), 8237 events (zygotes/two-cell stage), 5234 events (two-cell/four-cell stage), 2893 events (four-cell/eight-cell stage), and 4982 events (eight-cell stage/ICM). The differentially expressed genes numbered approximately 600 to 5000 depending on the comparison, while genes undergoing AS events numbered approximately 500 to 3000 (FDR < 0.05) (Fig. 2c). For both variables, the two-cell stage showed peak values, suggesting that there is an abundance of RNA AS at the two-cell stage.

### Alternative splicing at the two-cell stage

**Fig. 3:**
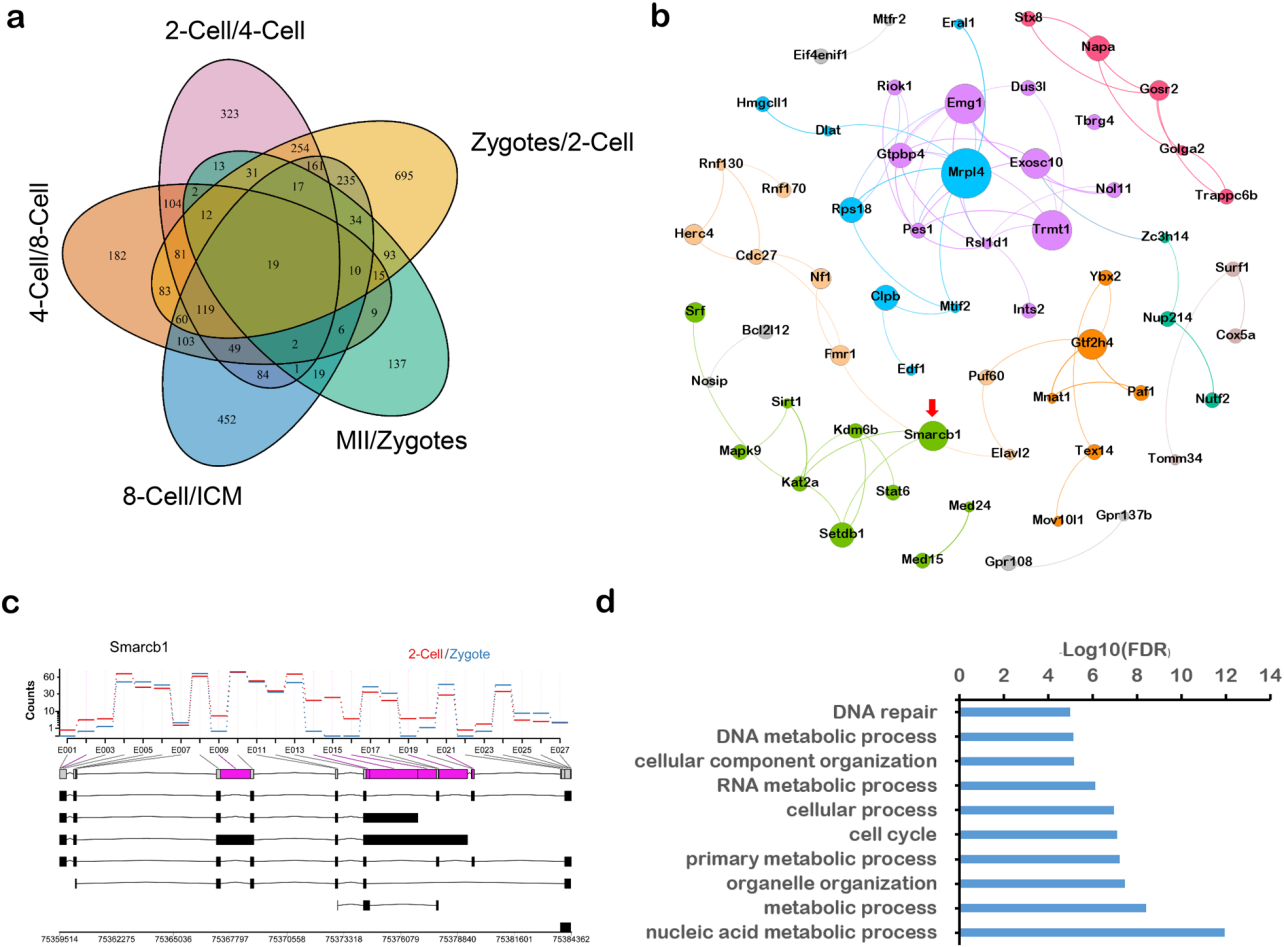
Alternative splicing at the two-cell stage. **a** Venn diagram shows the AS events in preimplantation embryo development. **b** Protein–protein interaction network of genes with AS only at the two-cell stage. **c** Visualization of Smarcb1 isoforms and expression in zygotes and at the two-cell stage. **d** Gene Ontology pathways of genes with AS only at the two-cell stage

To further study AS events at the two-cell stage, we identified 695 genes that exhibited AS in the two-cell stage compared with their expression in zygotes using a Venn diagram (Fig. 3a). We then input those genes into String (version 11.0) to create a protein–protein interaction (PPI) network. According to betweenness centrality, a measure representing the frequency of the shortest path in the network, we divided our network into nine modules (Fig. 3b). Among them, we particularly focused on *Smarcb1*, a sub-hub gene encoding an SWI/SNF-related matrix-associated actin-dependent regulator. It has seven isoforms in mouse and two in human, and this gene was previously reported to be related to chromatin remodeling [30–32]. Exon counts showed that most of the AS events occurred at exons 7–9,13–27, and 23–27, the latter of which correspond to the 3′ end of the CDS of SMARCB1 (Fig. 3c). In a previous study of Smarcb1 in human, it was reported that mutation in the C-terminal domain (CTD) of the Smarcb1 subunit could abrogate the DNA remodeling activity and accessibility, leading to changes of dominant gene regulation and morphology in human cells [30]. This in turn suggests that these different isoforms may play a role in the regulation of chromatin remodeling. Furthermore, we analyzed the enrichment of the genes exhibiting AS specifically at the two-cell stage using Gene Ontology and found that these genes were highly related to the DNA/RNA process and cell cycle (Fig. 3d). This indicates that the AS genes widely affect fundamental biological processes at the two-cell stage, perhaps in preparation for subsequent cell proliferation and differentiation.

### Difference of alternative splicing types in preimplantation embryo development

**Fig. 4:**
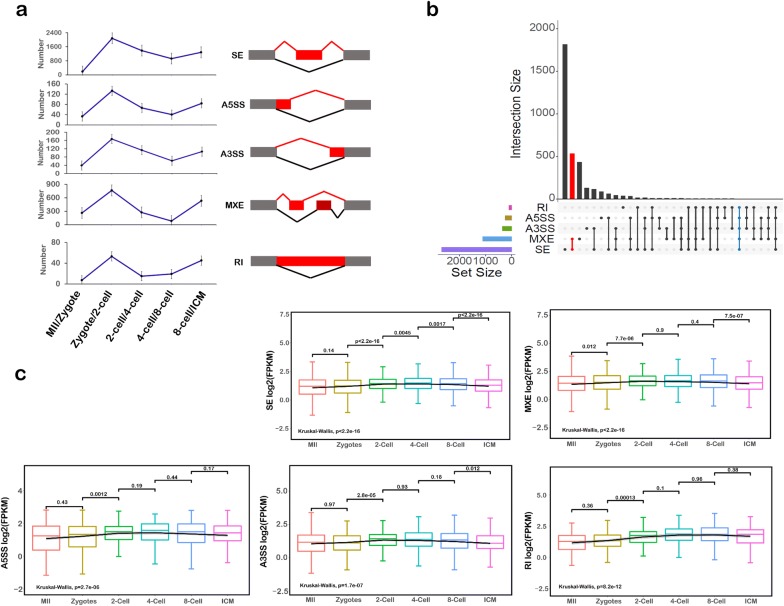
Difference of alternative splicing types in preimplantation embryo development. **a** Five types of AS genes in preimplantation embryo development. **b** Interactions between the five types of detected AS genes in preimplantation embryo development. **c** The expression of the five types of AS genes in preimplantation embryo development

By using rMATs based on the standard protocol as previously reported [33], we discovered 526 AS events in the MII/zygote stage, 3199 AS events in the zygote/two-cell stage, 1850 AS events in the two-cell/four-cell stage, 1132 AS events in the four-cell/eight-cell stage, and 2051 AS events in the eight-cell stage/ICM. According to their AS patterns, we roughly divided those AS events into five types: skipped exon (SE), alternative 5′ splice site (A5SS), alternative 3′ splice site (A3SS), mutually exclusive exons (MXE), and retained intron (RI) (Fig. 4a). We found that the SE and MXE types constituted large proportions of the AS events, with all the results being as follows: 186 SE events (MII/zygote), 2082 SE events (zygote/two-cell stage), 1382 SE events (two-cell/four-cell stage), 927 SE events (four-cell/eight-cell stage), and 1283 SE events (eight-cell stage/ICM), and 261 MXE events (MII/zygote), 764 MXE events (zygote/two-cell stage), 274 MXE events (two-cell/four-cell stage), 84 MXE events (four-cell/eight-cell stage), and 533 MXE events (eight-cell stage/ICM). The results show that both types of AS event were particularly abundant at the two-cell stage (Fig. 4a), supporting our previous conclusion that two-cell stage is important for AS events. Meanwhile, by overlap of each type of AS genes, we found two genes, the growth arrest-specific transcript 5 (*Gas5)* and *Psma3*, the former of which is a long noncoding RNA and the latter a coding gene (Fig. 4b). Interestingly, *Gas5* has more than 10 isoforms in the Ensembl database in mouse (visualized in UCSC genome browser). These many variants of *Gas5* suggest that this gene has a key role in preimplantation embryo development. Previous reports on *Gas5* described that it is related to the self-renewal and pluripotency of stem cells [34, 35], and it can also act as a competing endogenous RNA (ceRNA) to exert its functions. Furthermore, we found that the expression of AS genes was significantly different in the two-cell stage compared with other stages in preimplantation embryo development (Fig. 4c), also supporting our previous findings.

### Alternative splicing is linked to TAD boundaries at the two-cell stage

**Fig.5:**
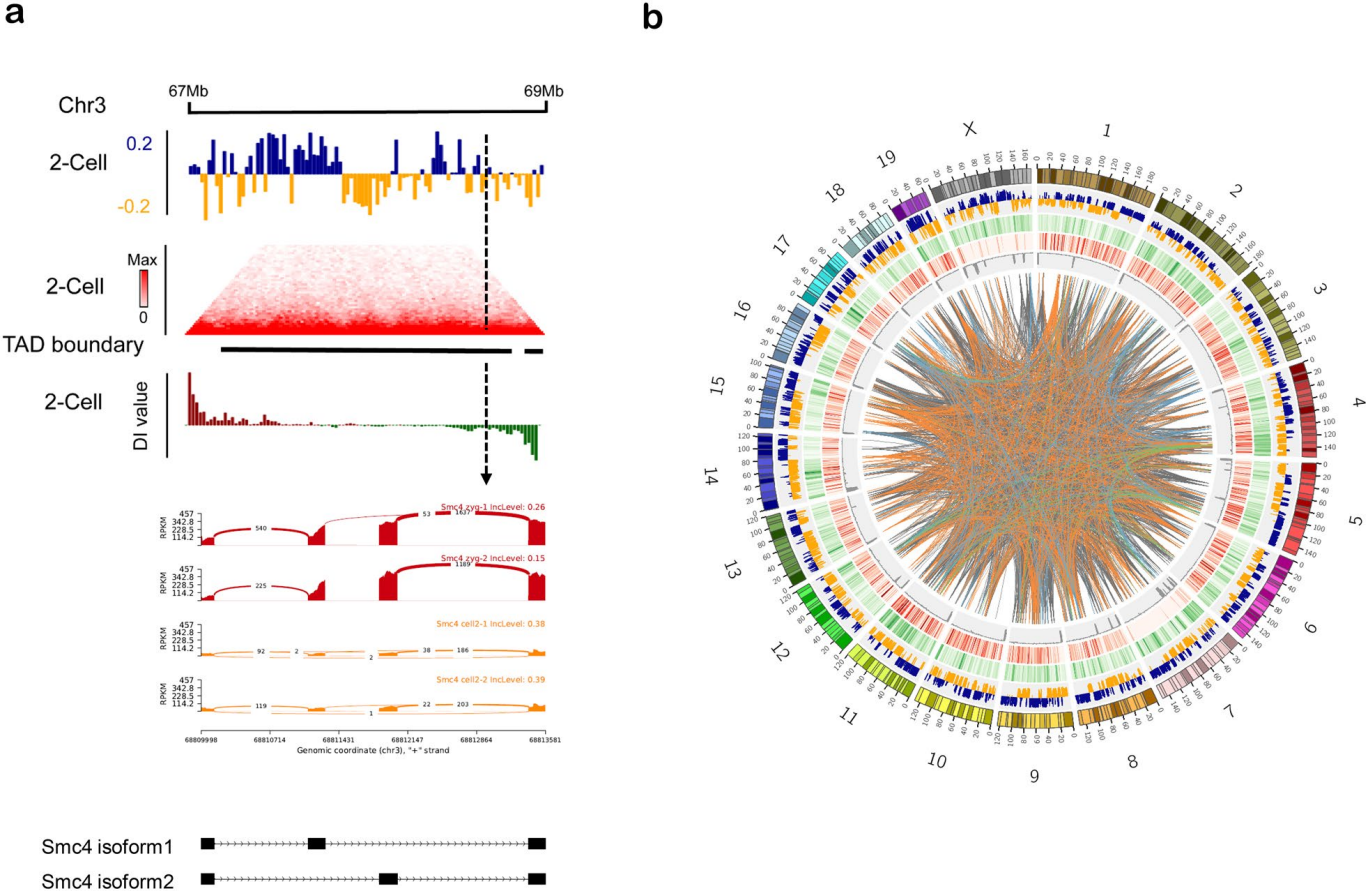
Alternative splicing is linked to the TAD boundaries. **a** Example of Smc4 isoforms at the TAD boundaries (top: blue represents A compartment, orange represents B compartment; middle: heatmap of normalized Hi-C data, TAD boundary region, and DI values; bottom: the expression of Smc4 isoforms in zygotes and at the two-cell stage). **b** Circos plot shows that the AS genes were particularly associated with the TAD boundaries. (the circles from outside to inside sequentially represent the A/B compartments, gene density (green), TAD boundaries (red), and IS scores (gray color); the links represent the protein network for different types of AS genes.)

The previous transcriptome data showed that the two-cell stage is a pivotal step in preimplantation embryo development. Meanwhile, the chromatin structure was also shown to be reprogrammed starting at this stage, as it was reported that TADs started to form, were subsequently strengthened, and their construction was completed at the eight-cell stage [11, 12]. To investigate whether the dynamic changes of chromatin organization were related to AS events, we analyzed the Hi-C data from the two-cell stage. We first measured the relationship between AS events and A/B compartments. By extracting the PC1 score from principal component analysis (PCA) [10], we found that AS events were not related to the A/B compartment status of chromatin structure (Fisher’sexact test, p = 0.1164). Then, we used the direction index (DI) method to call TADs at the two-cell stage [26]; Fisher’s exact test (p = 0.4466) showed that AS events were also not related to TADs. Lastly, we defined TAD boundaries as regions of less than 400 kb located between two TADs. We discovered 691 AS genes located in TAD boundaries, among the total of 3628 genes (Fisher’s exact test, p < 2.2e^−16^); the results showed that an abundance of AS events occurred in TAD boundaries. Figure 5a shows an example of Smc4 isoforms in the TAD boundaries (Fig. 5a). *Smc4*, structural maintenance of chromosomes (SMC) 4, belongs to the SMC gene family and is an important subunit of the cohes in protein complex. This cohesion has been reported to be related to the higher-order chromatin structure [36]. This suggested that AS events may play an important role in chromatin organization. Furthermore, to confirm that AS events were particularly abundant in TAD boundaries, insulation score (IS) was calculated to measure the strength of TAD boundaries; a higher absolute IS score indicated a stronger TAD boundary [37]. We then analyzed the frequency of AS events at the genome-wide scale (Fig. 5b). The circos plot showed that AS events were enriched at the TAD boundaries, but were not related to A/B compartments. Taken together, these results showed a link between AS events and chromatin organization.

## Discussion

Previous research has reported the AS genes expression in embryonic day 8.5, 9.5 and 11.5 in mouse using microarray [38]. They revealed AS events are frequent across developmental stages and tissues, proposing AS is an important developmental regulator [38]. However, it remains to be study the AS events in preimplantation embryo development because of technology limitation. With the development of scRNA-seq, the transcriptome profile analysis in preimplantation embryo development was successfully preformed [13, 39, 40]. However, they focused on the gene expression levels without distinguishing AS events during preimplantation embryo development.

AS plays a critical role in the regulation of gene expression and protein diversity in a variety of eukaryotes. In this study, we firstly identified the critical stage for gene transcription during the preimplantation embryo development to study signatures of AS in embryo development. By analyzing the time course of gene expression profile in preimplantation embryo development, we found the two-cell stage is a pivotal step in preimplantation embryo development because of the number of expressed genes. Then, we discovered that RNA splicing plays an important role in two-cell stage compared with zygote. Based on calculating PSI, we demonstrated that an abundance of AS events occurred in preimplantation embryonic development, with the two-cell stage having a high frequency of such events, the abundance of which then decreases at the four- and eight-cell stages, followed by a recovery at ICM. Our findings not only show that the two-cell stage is a pivotal step in preimplantation embryo development, but also indicate that AS events play an important role in the early embryo development.

One of the most important features in preimplantation embryo development is chromatin organization. In zygotes, the haploid genomes from paternal and maternal origins start to fuse, which greatly changes the chromatin structure. Previous reports indicated that organization of chromatin, as the structural and functional basis of the genome, can affect the location of DNA, playing important roles in gene transcription, preventing DNA damage, and ensuring DNA duplication and other biological processes [13, 41]. In this study, we found that AS events were particularly abundant at TAD boundaries. This may be because many transcription factors and histone modifications are related to TAD boundaries and the most important RNA polymerase, RNA Pol II, is also enriched in TAD boundaries [26]. At the TAD boundaries, we also identified that Smc4, a cohes in subunit protein, could form chromatin loops with CTCF to regulate the chromatin organization [42].

## Conclusions and perspective

In this study, we first showed that, within the stages of preimplantation embryo development, the two-cell stage had a higher number of expressed genes, suggesting the importance of this stage within this process. Based on that, we identified a cluster of genes that were highly expressed at the two-cell stage, which may be related to chromatin organization. Then, we discovered AS events in this stage and divided them into five types: A5SS, A3SS, MXE, SE, RI; our data showed that AS events were abundant in preimplantation embryo development, especially at the two-cell stage. According the profile of AS events in preimplantation embryo development, we found that most AS events were of the SE and MXE types, both of which frequently occurred at the two-cell stage. Lastly, we found that AS events were also related to chromatin structure, with them being particularly common in the TAD boundaries. Interestingly, some of the genes undergoing AS such as CTCF, Smc4, and Smarcb1 are all related to chromatin structure [43]. The findings provide insight into the relationships among gene expression, AS genes and high-order chromatin structure. These could form a self-regulatory loop between gene expression and chromatin organization, in which the transcription of some chromatin structure-related genes is initiated to promote the formation of TADs at the two-cell stage, and these well-constructed TADs then further promote the transcription of other cell development-related genes.

## Data Availability

The data and equipment used are presented in the manuscript.

## Abbreviations

scRNA-seq: Single cells RNA-seq
AS: Alternative splicing
TADs: Topologically associating domains
PSI: Percent spliced-in
ICM: Inner cell masses
CTD: c-Terminal domain
SE: Skipped exon
A5SS: Alternative 5′ splice site
A3SS: Alternative 3′ splice site
MXE: Mutually exclusive exons
RI: Retained intron
ceRNA: Competing endogenous RNA
PCA: Principal component analysis
